# Evaluating determinants of rural Villagers’ engagement in conservation and waste management behaviors based on integrated conceptual framework of Pro-environmental behavior

**DOI:** 10.1186/s40504-016-0045-3

**Published:** 2016-12-05

**Authors:** Piyapong Janmaimool, Chaweewan Denpaiboon

**Affiliations:** 1 Environmental Social Sciences Program, Department of Social Sciences and Humanities, School of Liberal Arts, King Mongkut’s University of Technology Thonburi, 126 Prachauthit Rd. Thungkru District, Bangkok, 10140 Thailand; 2 Urban Environmental Planning and Development Program, Faculty of Architecture and Planning, Thammasat University, Pathumtani Province, Thailand

**Keywords:** Conservation behavior, Environmental management, Pro-environmental behaviors, Waste management behavior

## Abstract

This study aims to evaluate determinants of villagers’ engagement in pro-environmental behavior (PEB), including ecological conservation behavior (ECB) and waste management behavior (WMB). An integrated exploratory model representing the proposed relationship between villagers’ engagement in ECB/WMB and their determinants was created based on the integration of the theory of planned behavior (TPB), the Stern’s value belief norm (VBN) theory, environmental education and psychosocial characteristic perspectives. The potential predictors included a community norm, environmental knowledge, sense of obligation and self-efficacy, life satisfaction, place attachment, environmental worldview, perceived local environmental values, and psychosocial characteristics. Questionnaire surveys with villagers residing in the Nernkhor sub-district, Rayong province, Thailand, were conducted. The results of multiple regression analyses revealed that individuals’ engagement in ECB and WMB could be predicted by a different set of predictors. ECB was well predicted by self-efficacy, place identity, and perceived environmental values; whereas, WMB was well predicted by community norm, gender, age, knowledge related to action strategies, and self-efficacy. Therefore, different environmental strategies should be constructed to enhance the engagement of villagers in pro-environmental behaviors.

## Introduction

Without proper development planning and strategies, human development activities have negative impacts on environmental quality and generate adverse consequences for natural ecological systems (Cui and Shi 2012; Haas et al. 2015; Habibullah et al. 2016). Haas et al. (2015) demonstrated that Shanghai, China is facing a decrease in natural areas as urban areas rapidly increase by approximately 120%. Cui and Shi (2012) revealed Shanghai’s cultivated lands have been replaced with construction sites, buildings and infrastructure. Consequently, the weather in Shanghai has significantly changed; namely, air temperature, the number of hot days has increased, and the relative humidity has decreased. Habibullaha et al. (2016) reported on the association of tourism activities with biodiversity losses in 141 countries. In Thailand, natural depletion is one of serious environmental problems. A researcher, Barbier (2007) revealed that Thailand faced extensive mangrove deforestation. In 2004, the area of mangrove forest estimated by Food and Agriculture Organization (FAO) was 2,500 km^2^, significantly less than the area of mangrove forest recorded in 1961, at 3,500 km^2^. In addition, Thailand is also facing massively increasing amounts of wastes. In 2015, the average amount of municipal solid waste was 26.85 million tonnes, higher than the amount of municipal solid waste recorded in 2004, at 22 million tonnes (Pollution Control Department, 2015). As a consequence of too much emphasis on income generation without the consideration of the existence of natural systems and environments, cities and/or local authorities face the potential risk of environmental degradation and loss of natural resource values. Governments in every country put much effort into protecting the environment from human development activities. Environmental responsibilities, however, should be laid with local communities and individuals as well since the way they live and consume resources significantly effects the environments in which they live.

Pro-environmental behaviors of rural villagers are essential to ecological conservation as well as environmental protection. Many researchers state that most environmental problems, including air pollution, drought, biodiversity degradation, and global warming, are rooted in negative human behaviors (DuNann Winter and Koger 2004; Vlek and Steg 2007). To eliminate those problems and/or to reduce those environmental impacts, human behaviors should be altered. The use of local natural resources, consumption, and waste disposal in the household are all defined as environmentally significant behaviors. Changes of those behaviors into more environmental friendly directions could bring some changes to local environmental quality and benefit natural ecological systems. Many people express a great environmental awareness (Pieters et al. 1998); however, the awareness does not contribute to individuals’ decision to engage in PEB. Reasons that an individual decides whether to perform PEB are complex and different, depending on the types of PEBs.

In Thailand, PEBs have been enthusiastically promoted but they have still not received much public attention, as evidenced by the degradation of ecosystems and the occurrence of environmental pollutions in some areas. Various human actives, such as overharvesting mangrove resources and clear-cutting of mangroves, have caused a rapid decrease in mangrove areas. Nernkhor sub-district, located in Rayong province, Thailand, is one of many areas which have plentiful natural resources, including water, mangrove forests, productive environments for agriculture, and a rich diversity of plant species. Those valuable resources benefit local villagers’ well-being in various aspects such as career, recreational activities, daily living, and health. The majority of the villagers’ careers are, therefore, related to the utilization of local natural resources as they work in agriculture and fishery. However, the utilization of those resources has its own limitations and requires effective management, especially since natural resource consumption has increased due to the increasing population. Furthermore, waste management should be taken into consideration to prevent the plentiful resources from being depleted and deteriorated.

This study aims to evaluate the determinants of pro-environmental behaviors of villagers, including ecological conservation behavior (ECB) and waste management behavior (WMB). The Nernkhor sub-district in Rayong was selected as a case study. The questionnaire surveys with 102 villagers were conducted in March 2016. The acquired data were inspected and analyzed by performing multiple regression analyses so as to test the influence of each predictor on villagers’ engagement in those two types of PEBs. The findings provide some implications for the encouragement of local villagers to partake in PEBs.

## Theoretical context

### Pro-environmental behavior (PEB)

Pro environmental behavior (PEB) is defined as actions that cause no harm to natural systems, and/or benefit the environment (Steg and Vlek 2009). PEBs are environmentally-friendly actions or environmentally responsible behavior and they can be classified into four categories: recycling and reusing behaviors, conservation behaviors, consumer behaviors, and transportation use behaviors (Schultz and Zelezny 1998). Winther et al. (1994) identified five major types of PEBs: environmental activism, non-activist political behaviors, sustainable consumer behaviors (e.g., purchasing environmentally friendly products, recycling products and reducing dirty energy use, and changing consumption behaviors), ecosystem behaviors or conservation behaviors, and other specific behaviors (e.g., reducing waste in manufacturing process, monitoring environmental impacts generated by industrial sectors, etc.). Some scholars include other relevant actions such as seeking environmental information, being involved in decision-making processes on environmental strategies, and valuing environmental stewardship (Hungerford and Volk 1990; Stern 2000). Among those categories, conservation behaviors and waste management behaviors, including recycling/reusing and waste reduction, are considered essential actions that tremendously influence local ecological systems. Conservation behavior refers to actions such as increasing the quantity of trees and biological species by planting various species of trees, protecting wildlife populations from adverse impacts of human activities, and avoiding recreational activities that impact ecological systems such as collecting plant seeds from natural areas (Hungerford and Volk 1990). The practices of these behaviors could ensure that ecological resources would be properly exploited and ecological areas would not be deteriorated by human activities. Finally, it is expected that both conservation behaviors and waste management behaviors contribute to sustainable ecological conservation in rural communities.

### Determinants of environmental behaviors

To investigate the potential factors determining individuals’ engagement in PEB, many scholars created a hypothetical model based on two major theories: the theory of planned behavior (TPB) and the Stern’s value belief norm (VBN) theory. TPB, first proposed by Ajzen 1991, indicates that individuals’ decisions to be involved in PEB are based on an individual’s behavioral intention, which is influenced by attitudes of the behavior, subjective norms, and perceived behavioral control (Ajzen 1991). Attitude refers to how an individual views and evaluates environmental behaviors. If PEB is positively evaluated by an individual, it is likely that he or she would have intention to engage in PEB. Social norms, referring to the individual’s perception of social pressure on the engagement in PEB, are also influential. Moreover, Ajzen stated that the decision to perform PEB is also dependent on perceived behavioral control. Namely, people will decide to act environmentally, if they think it is possible to act. Many scholars in the field of environmental psychology and environmental behaviors utilized TPB to explain why people conduct environmentally responsible actions (Corral-Verdugo and Armendariz 2000; Heath and Gifford 2006; Warner and Aberg 2006; Carmi et al. 2015). However, some scholars criticized the application of TPB to the investigation of individuals’ PEB due to its lack of moral judgment consideration (Kaiser et al. 2005). So as to comprehensively explain why people act environmentally, Stern (2000) proposed the VBN theory which assessed the influence of individuals’ moral judgment as well as perceived environmental values on decision to engage in PEB Stern (2000). The VBN theory became a popular theory with many scholars who used it to investigate determinants of individuals’ involvement in many types of environmentally responsible activities, such as energy saving actions (Bronfman et al. 2015; Testa et al. 2016), green consumption behaviors (Stern 2000), conservation behaviors (Susanne and Susanne 2010), and environmental policy support (Steg 2005). Additionally, some scholars argue that pro-environmental behavior is not only a function of moral and rational decision process as explained in TPB and VBN. Triandis proposed the Theory of Interpersonal Behavior (TIB) which includes emotive and habitual perspectives. Based on TIB, environmental behavior is influenced by four major factors; intention, affect, habit, and facilities (Gagnon et al. 2003). Some acts are not solely influenced by intention, but performed based on individual’s routinized behaviors. While some acts might be performed only in supportive situations such as conducting waste separation only when all types of bins are provided. Based on extensive reviews of the relevant literature, the potential determinants of rural villages’ PEBs can be classified into four major categories: social, dispositional and cognitive, attitudinal, and psychosocial characteristic.

#### Social factors

According to TIB and TPB, intention is considered as an important component of PEB. Intention means the individual’s motivation to practice PEB. Besides influenced by attitude of behaviors, motivation is tremendously influenced by social or community norms which are defined as a social factor (Liebrand et al. 1992). Community norms involve the social group’s shared expectations and beliefs about the ways community members should act (Thogersen 1999); they are the community’s rules or standards that are widely recognized by its members and that contribute to social behavior (Cialdini and Trost 1998). Many previous studies found a positive relationship between community norms and individuals’ engagement in PEBs (Bamberg and Schmidt 2003; Fornara et al. 2011; Matthies et al. 2012). If a community has a norm related to environmental protection, and that norm is recognized by members of the community, it is expected that individuals will partake in PEBs.

#### Dispositional and cognitive factors

Considering dispositional factors, it involves dispositional characteristics of an individual, which potentially influence PEB. According to TPB, feelings of self-efficacy, referring to individuals’ perception of the significance of their contribution to solving environmental problems (Sanchez 2010), has a strong influence on an individual’s motivation to engage in PEB. If an individual has a feeling of high self-efficacy, he or she is likely to make a rational decision to engage in PEBs. Besides self-efficacy, VBN theory also indicates the significance of personal moral norms on an individual’s predisposition to PEB. Sense of obligation or moral obligation, referring to individuals’ perceived responsibility to behave environmentally (Sanchez 2010), is considered as a personal norm. An individual, having high moral obligation, is more likely to partake in PEBs. Both self-obligation and self-efficacy were reported as significant predictors of PEBs in many previous studies (Ajzen and Fishbein 2000; Susanne and Susanne 2010; Chen 2015; Testa et al. 2016). Regarding cognitive factors, it involves individuals’ environmental awareness and motivation which is created based on individuals’ cognitive thinking process. In this sense, the cognitive factor refers to individuals’ acquisition of relevant environmental knowledge which is utilized for creating awareness and motivation to perform PEB (Grob 1995). Environmental knowledge encompasses several types of knowledge and skills, such as an individual’s capability to identify problems relevant to environmental systems, causes of environmental problems, as well as appropriate behaviors for environmental conservation and protection (Laroche et al. 2001). The study conducted by Bronfman et al. (2015) demonstrated that people who reported having high environmental knowledge were more likely to be involved in PEBs.

#### Attitudinal factors

Attitudinal dimensions refer to environmental attitudes and concerns which could affect PEBs. The attitudinal dimension concerns local environmental values, attitudes toward the environment, and place attachment. According to the Stern’s value belief norm (VBN) theory, environmental attitude is a key driver of PEB. When having positive environmental attitudes, individuals are likely to have a positive attitude towards PEB, which finally affects motivation to perform PEBs. To measure individuals’ environmental attitudes, environmental worldviews, defined as an individual’s views on the interconnection of humans and the natural environment, the benefits of limitations on development and economic growth, and humanity’s right to rule the rest of nature, were explored (Dunlap et al. 2000). Van Liere and Dunlap (1980). The scale aims to measure individuals’ perceptions of the relationship between nature and humans (Amburgey and Thoman 2012). Barr and Gilg (2006) demonstrated that people expressing a highly positive environmental attitude or environmental worldviews, reported a relatively positive attitude towards PEBs. Besides environmental worldviews, local environmental values perceived by individuals can also encourage them to engage in PEBs (Nordlund and Garvill 2002). The term "value" has diverse meanings in various disciplines (Brown 1984). Schroeder (2011) views environmental values as human experience in various kinds of beneficial outcomes generated by natural environments such as physical products, biological and physical outcomes, psychological and social outcomes as well as precious memory. Individuals who have a higher perception of environmental values may be more actively involved in PEBs than those who have a lower perception. Additionally, a positive relationship between place attachment and PEBs was found in many previous studies (Takahashi and Selfa 2015; Ramkissoon et al. 2012). Place attachment means the emotional and/or functional bonds that a person constructs or develops toward a particular place (Tuan 1974; Williams and Carr 1993). Place attachment can be classified into two distinct types: place dependence and place identity. Place dependence refers to a functional connection based on the individual physical connection to a place. It reflects the degree to which the physical setting provides conditions to support individuals’ life and well-being (Schreyer et al. 1981; Williams et al. 1992; Williams and Vaske 2003)**.** Place identity is constructed based on the combinations of feelings about particular physical settings and a symbolic relation to a place (Proshansky et al. 1983; Williams et al. 1992; Williams and Vaske 2003.) It is expected that individuals who have either place identity or place attachment are more likely to be engaged in PEBs in order to protect their living environments.

#### Psychosocial characteristic factors

Additionally, the degree of individuals’ engagement in PEBs is potentially influenced by psychosocial characteristic factors, such as life satisfaction, gender, age, education level, and income; these factors have been investigated in many previous studies related to environmental behavioral (Raudsepp 2001; Cottrell 2003; Shen and Saijo 2008; Xiao and Hong 2010; Kip Viscusi et al. 2011; Swami et al. 2011; Sanchez et al. 2016). The first factor is life satisfaction. People’s estimation of their life satisfaction may reflect an optimistic disposition. People who express high levels of optimism have a relatively high ability to cope with problems, while less optimistic people tend to avoid or ignore problems (Scheier et al. 1986; Rand 2009). Thus, it is possible that optimistic individuals will be more engaged in PEBs (Weber 2012). Gender and education level were often found as significant predictors of PEBs. Meyer (2016), for instance, demonstrated that individuals obtaining a higher education degree reported a higher level of engagement in PEBs. Sanchez et al. (2016) demonstrated that females reported a significantly higher level of involvement in green purchasing behavior than did males. Similarly, Xiao and Hong (2010) found that females reported more active engagement in PEBs inside of the home, such as in reusing and recycling activities, than men did. Additionally, the influence of individuals’ income on the prediction of PEBs was much investigated in previous studies, and the results showed a positive relationship (Shen and Saijo 2008; Poortinga et al. 2004; Lukman et al. 2013). Low-income people seemed to be reluctant to partake in PEBs because of financial difficulties (Ozaki 2011; Gadenne et al. 2011). Age, can be also a good predictor of PEBs; however the relationship between age and PEBs remains unclear because of conflicting results in previous studies. Raudsepp (2001) revealed that older people exhibited a higher level of environmental concern than younger people. Other studies, however, demonstrated that younger people had a greater sense of responsibility to the environment than older people did (Van Liere and Dunlap 1980; Dietz et al. 1998). Individuals’ periods of time living in a community is also a potential predictor because it could reflect a degree of relationship between people and their living environments. People living for a longer time in a community are expected to have a higher level of engagement in PEBs. All these socio-demographic factors were selected for investigation in this study.

### Theoretical framework

**Fig. 1:**
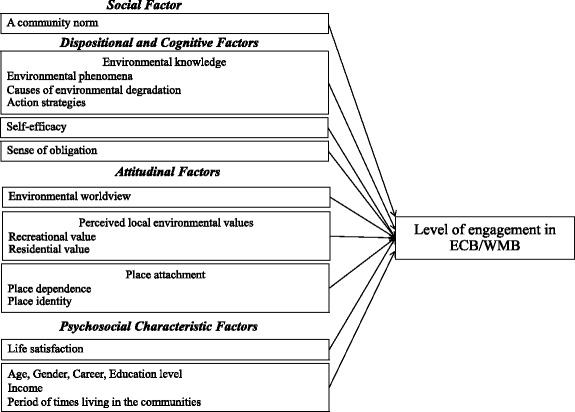
Study framework

Theoretical framework of this study was constructed in keeping with the findings from the literature review. The study aims to investigate determinants of PEBs of local villagers. Two types of PEBs, ECB and WMB, are selected. The frequency of individuals’ engagement in these two types of PEBs is defined as dependent variables. For the independent variables or potential predictors, the results of the literature review as well as primary surveys suggest four types of factors: social, dispositional and cognitive, attitudinal, and psychosocial characteristics. The proposed relationship between potential predictors and dependent variables is outlined in Fig. 1. The power of each variable in predicting each type of PEBs will be tested. The hypotheses of this study can be stated as follows:

- *Four types of selected factors, social, dispositional and cognitive, attitudinal, and psychosocial characteristics, can predict PEBs of local villagers.*
- *Predictors of ECB significantly differ from those of WMB*

## Methods

### Case study

**Fig. 2:**
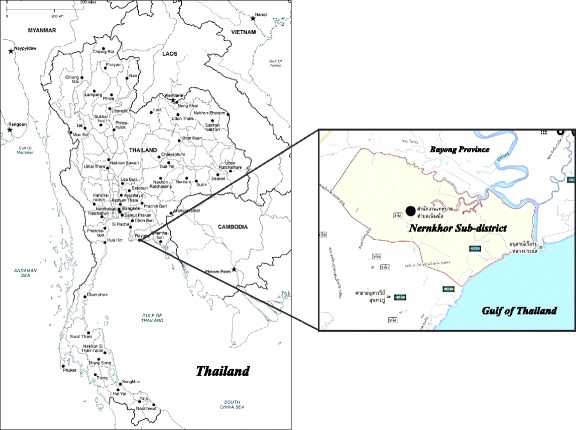
Study area

The Nernkhor sub-district of Rayong province is located near the coast in eastern Thailand (see Fig. 2). Its total area is about 36.08 km^2^. The area of mangrove forest is 1.7 km^2^, and 13.5 km^2^ is agricultural land; only 1.4 km^2^ is residential land (Nuenkhor Sub-district Administration. Thailand 2016). The population is 4,653 people or 1,251 households (Department of Provincial Administration. Thailand 2016). Nernkhor sub-district has plentiful natural resources such as the mangrove forest, areas with diverse species of domestic vegetables and fruits, and productive environments for agricultural activities. These resources provide villagers with a wide range of ecosystem services related to their careers and livelihoods. Most residents are agriculturalists and fishermen (Nuenkhor Sub-district Administration. Thailand 2016). According to the current situation, the mangrove ecosystem resources in this area are potentially threatened by human activities due to the increasing number of population and intensive harvesting of mangrove resources. The increasing number of population consequently increases the demand of mangrove resource exploitations. Without proper ecological practices carried out villagers, it is possible that these valuable resources cannot be sustainably exploited. Moreover, food manufacturing industries in the area also motivate local farmers to massively harvest aquatic animals for supplying them to the industries. In this way, effective ecological management measures are urgently required, and pro-environmental behaviors should be essentially promoted.

### Measurement of dependent variable

The study defines PEBs as dependents variables. ECB and WMB were explored by means of individuals’ self-reports. Individuals’ self-reports have been widely used by many scholars in the field of environmental behaviors (Warriner et al. 1984; Fujii et al. 1985). By using this method, participants will be asked to indicate the frequency with which they engage in each specific type of PEBs (Gatersleben et al. 2002). The authors developed five-point Likert scale questions (see Table 1). For the measurement of individuals’ engagement in ECB, respondents were asked to indicate the level of engagement in the biological conservation activities such as avoiding cutting trees, planting trees in damaged forest areas, or conducting integrated farming, and also to indicate whether they have harvested baby crabs and/or pregnant crabs. For WMB, the level of engagement in reusing and recycling activities and waste reduction acts were measured. The answers were measured on a scale from 1 (No involvement) to 5 (Regular involvement). The data gained from the questions related to ecological conservation would be added and calculated as a mean score, representing the degree of engagement in ECB. Like the calculation of the ECB score, the WMB score would be generated by calculating a mean score from the data gained from questions related to waste management.

**Table 1:** Factors, variables, and development of questionnaire

Factors	Variables	Survey questions	Response categories
Pro-environmental behaviors: ecological conservation behaviors (ECB)	Frequency of engagement in - Biological conservation - Ecological conservation such as local animal conservation	Have you ever been involved in the community’s environmental conservation activity such as avoiding cutting trees, planting trees in damaged forest areas, or conducting integrated farming?	1 = No involvement 5 = Regular involvement
		Have you ever eaten or harvested baby crabs and/or pregnant crabs caught in the community?	1 = Regular involvement 5 = No involvement
Pro-environmental behaviors: Waste management behaviors (WMB)	Frequency of engagement in - Reusing and recycling products - Waste reduction	How often do you reuse or recycle things such as plastic bags and bottles?	1 = No involvement 5 = Regular involvement
		Have you avoided using a plastic bag when buying a few goods?	
Life satisfaction	Satisfaction with life overall	Are you satisfied with your life in general?	1 = Not at all 5 = Absolutely
	Satisfaction with social condition	Are you satisfied with the social condition in the community?	
	Satisfaction with economic status	Are you satisfied with your economic condition?	
Place attachment	Place dependence	I feel comfortable living in this community.	1 = Absolutely not 5 = Absolutely yes
	Place identity	I am very attached to this community.	
Environmental worldview	Perception of “human-nature relation”	How much do you agree with these statements? -The balance of nature is very fragile and easily disrupted. -Human activities sometimes contribute to environmental changes. -Human beings have the right to modify the environment in response to their needs. -The ecological system exists to support human needs. -Development has its limitations, and population growth is higher than nature can support.	1 = Strongly disagree 5 = Strongly agree
	Perception of “limiting growth”		
	Perception of “human dominance over nature”		
Perceived environmental values	Perceived environmental values	Your community has fresh air and good environments suitable for recreational activities.	1 = Strongly disagree 5 = Strongly agree
		Your community has fresh air, good environments suitable for living.	
Environmental knowledge	Environmental phenomena	How much do you know about current environmental phenomena such as the climate situation?	1 = Not at all 5 = Well understand
	Causes of environmental problem	How much do you know about the cause of environmental problems such as climate change?	
	Environmental management	How much do you know about how to act environmentally?	
Sense of obligation and self-efficacy	Willingness to pay for the environment	Are you willing to pay more for environmentally friendly products?	1 = Absolutely not 4 = Absolutely willing
	Perception of the self’s capability to solve environmental problems	Do you think a single person’s actions can contribute to the improvement of environmental quality?	1 = Absolutely not 5 = Absolutely contribute
Social norm	Social norm	Your neighbors pay attention to issues related to ecological conservation and environmental protection.	1 = Not at all 5 = Absolutely
Psychosocial characteristics	Gender	Please indicate your gender	
	Age	Please indicate you age	
	Career	Please indicate your career	
	Education level	Please indicate your education level	
	Income	Please indicate your income/month	

### Measurement of independent variables

There are four types of independent variables: social, dispositional and cognitive, attitudinal, and psychosocial characteristic variables. The first group involves the community norms. To measure individuals’ recognition of community norms related to environmental and ecological protection, the authors developed a five point Likert scale question. Respondents were asked to indicate the degree to which other community members pay attention to the conservation of natural environments. The scale ranged from 1 (Not at all) to 5 (Absolutely). The second group is dispositional and cognitive factors; it includes the variables of environmental knowledge, sense of obligation and self-efficacy. The study defined three types of environmental knowledge that possibly influence individuals’ environmental awareness and motivation to act environmentally. That knowledge includes the current environmental situation, causes of environmental degradation, and environmental strategies (Hines et al., 1986). To measure respondents’ acquisition of the abovementioned environmental knowledge, respondents were asked to indicate the degree of their understanding of the general environmental situation such as climate change, root causes of environmental degradation, and individuals’ environmental strategies. The scale ranged from 1 (Do not understand at all) to 5 (Well understand). For the measurement of sense of obligation and self-efficacy, the questions developed by Sanchez (2010) were adapted. Sanchez (2010) suggests that individuals’ expression of their acceptance of “paying more money to purchase green products or services for environmental and ecological protection” can reflect individuals’ sense of obligation. The authors, therefore, developed a Likert scale question by asking whether respondents are willing to pay more money to purchase green goods. The answers were measured on a scale from 1 (Absolutely not) to 5 (Absolutely willing). To measure self-efficacy, respondents’ realization of their significant role in solving environmental problems was examined. Participants were asked to indicate the level of agreement on the significance of their contribution to eliminating environmental problems. The scale ranged from 1 (Absolutely not) to 5 (Absolutely contribute).

The third group is attitudinal factors: perceived local environmental values, attitudes toward the environment, and place attachment. Perceived environmental values are defined as individuals’ perception of the significance of ecosystem services provided by local environments (Paelkhe 1994). Based on the definition, the authors developed a question asking what people think about the contribution of local environments to the community’s livable environments for living and recreational activities, and the scale ranged from 1 (Strongly disagree) to 5 (Strong agree). Regarding individuals’ attitude towards environments, the NEP scale, used by many scholars to measure individuals’ environmental attitude (Corral-Verdugo and Armendariz 2000; Ahlheim et al. 2013; Atava et al. 2015), was adapted to explore villagers’ perception of three important dimensions: human-nature connection, limiting growth, and human dominance over nature. Respondents were asked to express their level of agreement with the five statements (see Table 1). The last variable in the attitudinal group is place attachment, which is divided into two aspects: place dependence and place identity. According to the definitions stated in Theoretical framework section, the authors developed questions for measuring these two variables. For the measurement of place dependence, respondents were asked about their feeling of comfort when living in the community. To measure place identity, respondents were asked to indicate their feeling of connectedness. The answers of those questions were measured on a scale from 1 (Absolutely not) to 5 (Absolutely yes). The last independent variable group is psychosocial characteristic factors, which include life satisfaction, gender, age, educational level, period of time living in the community, and income. To measure individual life satisfaction, this study employed the life satisfaction questions proposed by World Values Survey (WVS) (Inglehart et al. 2004). Respondents’ subjective judgments on aspects relevant to their life, such as satisfaction with the social environment, satisfaction with their own economic condition, and satisfaction with life in general, were explored. The answers were measured on a scale from 1 (No satisfaction) to 5 (Very high satisfaction). The results obtained from those questions would be calculated as a mean score, representing a level of life satisfaction. Finally, to collect others socio-economic data for variable measurement, respondents were simply asked to indicate the required information on the questionnaire sheet.

### Data collection and analysis

The sampling group of this study was rural villagers living in the communities in Nernkhor sub-district. The authors distributed 150 questionnaire sheets to the sampling groups. However, only 102 questionnaire sheets were completed and deemed suitable for data analysis. Some questionnaire sheets were not fully completed and some of the sampling groups did not reply because some of rural villagers are illiterate. To receive a high response rate, the authors read and explained all the questions for some specific groups such as the elderly and illiterate respondents, and then they were asked to indicate their answers.

The quantitative analyses were performed to analysis of determinates of PEBs. The data gained from questionnaire surveys were statistically analyzed. First, the internal consistency of the scales developed for variable measurements was evaluated by calculating the Cronbach’s alpha. In this study, the Cronbach’s alpha was 0.826, which was above the required 0.7. Therefore, it can be concluded that the data gained from the questionnaire surveys were reliable. Then, the authors performed multiple regression analyses so as to test the proposed relationship between potential predictors and PEBs. The results of the analyses would demonstrate determinates of rural villagers’ engagement in PEBs, including ECB and WMB.

## Result and discussion

### Ecological conservation and waste management behaviors of rural villagers and descriptive statistics of predictors

The survey results revealed that rural villagers reported a higher level of engagement in conservation behaviors than in waste management behaviors. Table 2 depicts that an average ECB score was 3.6 with a standard deviation of 0.9, whereas an average WMB score was 2.5 with a standard deviation of 0.8. The number of female respondents was slightly higher than that of male respondents at 56.3 and 43.8%, respectively. The average age of respondents was 47 with a standard deviation of 14.8 years. Almost 46% of respondents have a primary school degree. Respondents with high school degree and university degree accounted for 26.5 and 27.5% respectively. The majority of respondents, approximately 51%, were gardeners and fishermen, and 16.7% were merchants and personal businessmen. The number of laborers accounted for 16.7%, and almost 10% were from governmental offices. The number of student respondents accounted for only 5.8%. The survey result also revealed that the average income of respondents was 19,200 Baht, and the average period of time living in the community was 39.3 years.

**Table 2:** Average ECB and WMB scores, descriptive statistics of potential predictors, and Cronbach’s alpha

Items	Mean/N	±/%	Cronbach' s α
ECB 1: Involvement in biological conservation activities	3.6	±1.0	.814
ECB 2: Involvement in ecological conservation activities	3.3	±1.2	.818
Total ECB score	3.6	±0.9	-
WMB 1: Involvement in reusing and recycling products	3.0	±1.0	.813
WMB 2: Involvement in waste reduction behavior	1.9	±1.1	.818
Total WMB score	2.5	±0.8	−
Gender			−
Male	45	43.8%	
Female	57	56.3%	
Age	47	±14.8	−
Education			−
*Primary school degree*	47	46.1%	
*High school degree*	27	26.5%	
*University degree*	28	27.5%	
Career			−
*Gardener/Fisherman*	52	51.0%	
*Merchant/Personal businessman*	17	16.7%	
*Governmental officer*	10	9.8%	
*Student*	6	5.8%	
*Laborer*	17	16.7%	
Income	19,200.7	±3,961.5	−
Period of time living in the community	39.3	±16.5	−
Community norm	3.7	±1.1	.821
Knowledge related to environmental phenomena	3.7	±0.8	.816
Knowledge related to causes of environmental degradation	3.5	±0.8	.817
Knowledge related to action strategies	3.4	±0.9	.814
Self-efficacy	3.7	±0.7	.821
Sense of obligation	2.8	±1.0	.826
Perceived local environmental values/recreational value	4.5	±0.6	.825
Perceived local environmental values/residential value	4.4	±0.7	.825
Environmental worldview			−
*The balance of nature is very fragile and easily disrupted.*	3.8	±0.7	.826
*Human activities sometimes contribute to environmental changes*	3.7	±0.9	.824
*Human beings have the right to modify the environment in response to their needs*	3.8	±0.8	.813
*The ecological system exists to support human needs.-*	3.6	±1.0	.817
*Development has its limitations, and population growth is higher than nature can support*	3.8	±1.0	.821
Average total score of environmental worldview	3.7	±2.0	−
Place dependence	4.1	±1.0	.819
Place identity	4.2	±1.1	.819
Life satisfaction			
*Satisfaction with life overall*	3.9	±0.9	.816
*Satisfaction with social environment in the community*	4.1	±0.9	.816
*Satisfaction with income-expenditure balance*	3.5	±1.1	.821
Average mean score of life satisfaction	3.8	±0.8	−

*N = 102*

Considering descriptive statistics of social, psychological and cognitive, and attitudinal variables and the value of Cronbach’s alpha, which presents reliability of measurement, those variables showed good reliability with Cronbach alphas all higher than 0.70 (George and Mallery 2003). Community norm has an average score of 3.7 (SD **=** 1.1) with a Cronbach alpha of 0.821. Compared to other types of environmental knowledge, knowledge related to environmental phenomena has the highest mean score 3.7 (SD **=** 0.8) with a Cronbach alpha of 0.816. Knowledge related to causes of environmental problems had an average score of 3.5 (SD **=** 0.8), and Knowledge related to action strategies had an average score of 3.4 (SD **=** 0.9). Self-efficacy showed an average of 3.7 (SD **=** 0.7). The respondents also reported a low sense of obligation with a total average of 2.8 (SD **=** 1.0). The Cronbach alpha values for these two scales are above 0.80. Perceived environmental values in supporting recreational activities and residential environments were high, 4.5 (SD **=** 0.6) and 4.4 (SD **=** 0.7) respectively. Environmental worldview had an average total score of 3.7 (SD = 1.1). Before being calculated as an average total score, the total scores of environmental worldview were derived by adding together positive variables (WV1, WV2, and WV5) and deducting two negative variables (WV3 and WV4). Respondents also reported high place dependence with a total average score of 4.1(SD **=** 1.0), and high place identify with a total average score of 4.2 (SD **=** 1.0). The Cronbach alpha values for two types of place attachment scales are above 0.80. Life satisfaction has an average mean score of 3.8 (SD = 0.8). Compared to other life satisfaction variables, satisfaction with social environment in the community was the highest score 4.1 (SD **=** 0.8) with a Cronbach alpha of 0.816.

### Factors determining ECB and WMB

To test the influence of the independent variables on rural villagers’ engagement in ECB and WMB, multiple regression analyses were performed. The results are shown in Table 3 below. The multiple regression model for predicting ECB is significant with *F* (18, 83) = 10.216, *p* = 0.000. The multiple correlation coefficient (R) was 0.830, and R square was 0.689. This indicates that approximately 68.9% of the variance in ECB can be accounted for by the linear combination of those selected predictors. There is no multicollinearity as a result of the variance inflation factor (VIF); the test showed the VIF values were between 1.271 and 3.540. They are all below the threshold value of 10 (Field, 2009). Similarly, the model for predicting WMB is also significant with *F* (18, 83) = 5.957, *p* = 0.000. The multiple correlation coefficient (R) was 0.751, and R square was 0.564. Approximately 56.4% of the variance in WMB can be accounted for by the linear combination of selected independent variables. However, when considering the potential of each selected independent variable in predicting both types of PEBs, it was found that ECB and WMB could be predicted by different significant predictors. ECB was significantly predicted by five predictors: self-efficacy, place identity, perceived local environmental values in providing livable residential environment, knowledge related to causes of environmental degradation, and period of time living in the community. Among those five significant predictors, self-efficacy is the most significant variable with a coefficient of 0.366. Place identity and perceived local environmental values in providing livable residential environment are significant at 0.1%, and their coefficient values are 0.359 and 0.251 respectively. Knowledge related to causes of environmental degradation and period of time living in the community are significant at 1%; their coefficient values are 0.221 and 0.195 respectively. In the case of the significant predictors of WMB, it was found that a community norm and gender are the most significant variables with equal coefficients of 0.303 and 0.278 respectively. Knowledge related to action strategies, age, and self-efficacy are significant at 0.5%, and their coefficient values are 0.324, 0.206, and 0.281 respectively. Notably, no attitudinal variable could predict WMB.

**Table 3:** Summary of regression analysis for variables predicting ECB and WMB

Factor	Variable	Determinants of ECB				Determinants of WMB			
		B	SE B	β	VIF	B	SE B	β	VIF
Psychosocial characteristic factors	Gender	−.052	.140	−.031	1.805	.467	.163	.278***	1.805
	Age	.002	.006	.026	2.451	.016	.006	.281**	2.451
	Education level	.025	.080	.025	1.659	.121	.093	.122	1.659
	Career	−.046	.037	−.089	1.335	−.014	.043	−.028	1.335
	Income	−2.854E−06	.000	−.048	1.539	−8.023E−06	.000	−.136	1.539
	Period of time living in the community	.010	.006	.195*	3.137	−.005	.007	−.106	3.137
	Life satisfaction	.090	.080	.087	1.592	.001	.094	.001	1.592
Social factor	A community norm	.093	.056	.122	1.446	.228	.066	.303***	1.446
Dispositional and cognitive factors	Knowledge related to environmental phenomena	−.173	.119	−.164	3.368	−.043	.139	−.042	3.368
	Knowledge related to causes of environmental degradation	.241	.125	.221*	3.540	.153	.146	.143	3.540
	Knowledge related to action strategies	.006	.105	.006	3.139	.310	.123	.324**	3.139
	Self-efficacy	.445	.084	.366***	1.271	.247	.098	.206**	1.271
	Sense of obligation	.035	.071	.039	1.732	−.054	.084	−.062	1.732
Attitudinal factors	Perceived local environmental values/recreational value	.115	.120	.078	1.773	.066	.140	.045	1.773
	Perceived local environmental values/residential value	.308	.098	.251***	1.712	.089	.115	.074	1.712
	Environmental worldview	−.009	.035	−.022	1.961	.015	.041	.037	1.961
	Place dependence	−.029	.066	−.035	1.729	.057	.077	.070	1.729
	Place identity	.282	.072	.359***	2.267	−.082	.085	−.106	2.267
Statistics		*R* ^*2*^ = 0.689; Adjusted *R* ^*2*^ = 0.622				*R* ^*2*^ = 0.564; Adjusted *R* ^*2*^ = 0.469			

*N = 102*

*p <0.1

**p <0.05

***p <0.01

The findings of determinant analyses provide a basic understanding on how villagers engaged in ECB and WMB. Notably, two types of PEBs could be predicted by a different set of independent variables. Considering ECB, place identity and self-efficacy played the most important role in predicting villagers’ engagement in ECB; another highly significant variable was perceived environmental value. In Nernkhor sub-district, villagers’ well-being and life styles are strongly connected with the mangrove resources. Several ecological services provided by the mangrove forests benefit villagers in various aspects such as recreational activities, career, daily living, and health. Many villagers also perceive the mangrove forest as the communities’ identity which must be conserved. Therefore, when feeling concerned with the deterioration of the mangroves, villagers would be eager to engage in ECB. This finding is related to the study of Mullendore et al. (2015), which stated that place identity greatly influences a wide range of conservation behaviors of U.S. farmers, such as conservation program enrollment, adoption of buffers and grassed waterways. It could be argued that when people identify the community as their house, they prefer to keep their house clean and livable. Self-efficacy also has a significant effect on ECB. This result confirms the use of TPB to explain PEBs (Ajzen 1991). Villagers will act environmentally if they perceive that their acts contribute to desired ecological outcomes (Sheeran and Abraham 2003; Sawitri et al. 2015). On the other hand, individuals who lack self-efficacy mostly believe that environmental problems can be successfully solved by other parties such as governments (Kollmuss and Agyeman 2002), thus they hesitate to act environmentally. To encourage rural villagers to engage in ECB, communication strategies should include the factor of self-efficacy. Villagers’ perceived environmental value is also important. The result of statistical analysis showed that villagers with higher perception on environmental values in providing livable residential environments relatively reported engagement in ECB with a significantly higher level. This finding implies that villagers’ satisfaction with the quality of local environments, and reported highly perceived environmental values, might have more awareness of threats to their living environments; thus, they are more active in ECB (Nordlund and Garvill 2002).

In addition, it was found that general environmental knowledge was not a strong predictor of ECB. Many types of environmental knowledge did not have a significant effect on villagers’ decision to engage in ECB. The result of statistical analysis demonstrated that only knowledge related to causes of environmental degradation had a significant effect on ECB. This implies that if villagers understand the root causes of environmental problems, they might have more environmental awareness and have more environmental responsibility. Thus, they potentially decide to engage in ECB. Therefore, communicating this kind of knowledge could enhance villagers’ engagement in ECB. Period of time living in the community is also an issue since the result showed that villagers with living longer in the area were more active in ECB. New residents of Nernkhor sub-district might not deeply understand the relationship between mangrove system resources and rural villagers’ cultures and life styles. Those villagers with living shorter in the area consequently engaged in ECB with a significantly lower level. Therefore, it could be suggested that more attention should be paid to younger or new residents to promote ECB in rural communities. Environmental communication strategies should be specially designed for this group.

Considering rural villagers’ engagement in WMB, the results revealed a different set of independent variables. Community norm and gender were the most powerful predictors of WMB. In the Thai rural society, a social norm seems to be a very important factor which influences members’ behaviors. WMB of villagers in Nernkhor sub-district was also significantly influenced by villagers’ perceived social pressure. It is possible that villagers would perform WMB, if general members of a society view that WMB is a good practice exhibiting individual sense of environmental responsibility. Villagers who hesitate to practice WMB can be negatively viewed by a general public. Many villagers in Nernkhor sub-district might be afraid of a negative perception from a general public; thus, they decided to engage in WMB. This finding correlates with the theory of planned behavior that states that PEB is potentially influenced by the social pressure (Ajzen 1991). Similarly, Thomas and Sharp (2013) found a positive relationship between social norms and recycling behaviors. Whether to act environmentally depends on the general expectations of other people in a society. Additionally, it was found that women reported a significantly higher level of engagement in WMB than men did. This finding corresponds to Meyer (2016) study, which revealed that female participants were more active in recycling and double-sided printing behaviors. Similarly, the result of an investigation by Xiao and Hong (2010) revealed that women reported higher engagement in domestic PEBs than men did. Women might have more concerns on issues related to their lives, thereby impacting their actions. Besides gender, the age of villagers also influenced WMB.

Regarding the results of previous studies, the influence of age on PEB is inconsistent. A positive relationship between age and PEB was found by Raudsepp (2001), but the result of an investigation conducted by Dietz et al. (1998) revealed a negative relationship. However, the result found in this study suggests that younger villagers’ engagement in WMB needs to be enhanced and proper communication strategies should be designed for this group. Younger villagers in Nernkhor sub-district might have low local environmental awareness due to insufficient understanding of the connection between community’s well-being and the natural environments; thus, they might not recognize the significance of engagement in WMB. Environmental knowledge related to action strategies also had a significant effect on WMB; whereas, other types of environmental knowledge were not significant predictors. Therefore, educating rural villagers with this type of knowledge may increase their willingness to participate in WMB. As stated by Stern (2000), lack of adequate environmental action knowledge hinders individuals from performing appropriate waste managing behaviors. The last predictor of WMB is self-efficacy, the only variable that could predicted both ECB and WMB. Self-efficacy could bridge the gap between environmental awareness and the decision to engage in PEB (Steg 2005; Marcle 2013; Chen 2015). Individuals will decide to engage in environmentally responsible behaviors when they realize that their actions contribute to environmental quality improvement to some extent. To increase rural villagers’ self-efficacy, environmental communication with specific information, such as the significance of WMB and ECB in environmental quality management, should be promoted.

Overall, this study provides some guidance for the development of environmental strategies which can promote pro-environmental behaviors of rural villagers in Nernkhor sub-district. To widely promote villagers’ engagement in ECB, new residents’ place attachment, particularly perceived place identity, must be enhanced. Place identity is the individual’s direct feelings or memories about a particular place (Williams and Vaske 2003). To create the perception of place identity, new residents should be encouraged to frequently participate in community activities. This will allow new residents to construct some memories about the place and relevant stories. In addition to place identity, environmental knowledge related to the causes of environmental problems can effect villagers’ motivation to engage in ECB; therefore, communicating this kind of knowledge potentially promotes ECB. Considering the promotion of WMB in Nernkhor sub-district, community norms might be used as a tool. Villagers’ understanding of the community norms concerning the local environments and WMB should be enhanced and widely promoted. This is because villagers are aware of the public expectation of their behaviors. In addition, providing rural villagers with environmental knowledge related to individual waste management action strategies can also increase villagers’ capability to perform WMB, and consequently contributes to the motivation to engage in WMB.

## Conclusion

In this study, determinants of ECB and WMB were examined by means of multiple regression analyses. The integrated framework for the investigation of PEBs was created based on relevant theories such as the theory of planned behavior (TPB), the Stern’s value belief norm (VBN) theory, environmental education and psychosocial characteristic perspectives. The results of multiple regression analyses revealed that rural villagers’ engagement in ECB and WMB could be predicted by a different set of predictors. ECB was significantly predicted by self-efficacy, place identity, perceived environmental values in providing livable residential environment, knowledge related to environmental degradation, and period of time living in the area. While, WMB was significantly predicted by a community norm, gender, age, knowledge related to environmental action strategies, and self-efficacy. The study suggests that to enhance rural villagers’ engagement in different types of PEBs, different environmental strategies should be constructed based on their potential determinants. The results of this study might be applied to promote pro-environmental behaviors in other Thai rural communities where have similar social and physical contexts as Nernkhor sub-district, Rayong province Thailand.

## Limitation of the study

Although this study has provided basic understandings on how to promote pro-environmental behaviors of rural villagers in the Thai rural communities, the results might not be proper to be generalized to the general rural population due to the small sampling size. Therefore, a future research which can engage a larger number of sampling populations is recommended.
